# Motives for Studying and Student Wellbeing: Validation of the Motivational Mindset Model

**DOI:** 10.3389/fpsyg.2021.753987

**Published:** 2021-12-03

**Authors:** Job Hudig, Ad W. A. Scheepers, Michaéla C. Schippers, Guus Smeets

**Affiliations:** ^1^Erasmus School of Social and Behavioral Sciences, Erasmus University, Rotterdam, Netherlands; ^2^Rotterdam School of Management, Erasmus University, Rotterdam, Netherlands

**Keywords:** mindset, purpose in life, student engagement, wellbeing, gender, ethnicity

## Abstract

Research on the joint effect of multiple motives for studying was recently given a push in a new direction with the introduction of the motivational mindset model (MMM). This model contributes to a better understanding of study success and student wellbeing in higher education. The aim of the present study is to validate the newly developed model and the associated mindset classification tool (MCT). To this end, 662 first-year university students were classified in one of the four types of motivational mindset using the classification tool and three exploratory validation procedures were conducted through sense of purpose, study engagement, and students’ background characteristics in terms of gender and ethnicity. Both purpose and study engagement are central dimensions of student wellbeing and predictors of study success. The results show that (1) sense of purpose and study engagement differ across the four types of mindset, (2) students in the low-impact mindset show the least optimal pattern of study engagement and sense of purpose, (3) sense of purpose and study engagement are positively related and this relationship is consistent across mindsets, and (4) overall differences in purpose and study engagement between gender and ethnic subgroups stem from one specific type of motivational mindset. The results provide support for the validity of the MMM and the usefulness of the MCT. The implications of the findings are discussed as well as promising avenues for future research.

## Introduction

Motives for studying, or the reasons why students go to university, are related to a wide array of important educational outcomes, such as wellbeing (Yeager et al., 2014), retention (Wang et al., 2009), adjustment to the university environment (Dennis et al., 2005; Kennett et al., 2013), and grades (Côté and Levine, 1997; Dennis et al., 2005; Kennett et al., 2013; Yeager et al., 2014). While research on motives for studying assumes that students hold multiple motives for studying simultaneously to varying degrees (Côté and Levine, 1997; Henderson-King and Smith, 2006), existing studies have analyzed students’ reasons for going to university mainly from a variable-centered or dimensional perspective. This approach treats motives for studying as independent variables, which misses out on the possibility to test the joint effect of multiple motives that students may have. Given the powerful influence of students’ motives on their studies and study environment, examining the interplay between motives is key to (a) better explain student differences in study success and wellbeing and (b) to understand different effects that interventions can have in terms of study success and wellbeing.

In a recent exploratory study, Hudig et al. (2020) therefore followed a student-centered, multidimensional approach by combining several motivational dimensions which have been shown to be importantly related to academic functioning and wellbeing (Yeager et al., 2014). They investigated the naturally occurring interactions between three dimensions of motives for studying: (1) intrinsic self-transcendent motives (i.e., studying to make the world a better place), (2) intrinsic self-oriented motives (i.e., studying because it is personally interesting and enjoyable), and (3) extrinsic self-oriented motives (i.e., studying to acquire a separable outcome, such as money or making new friends). Using cluster analysis, Hudig et al. (2020) identified four meaningful motivational profiles in two large cohorts of first-year students and coined the term *motivational mindsets*, explicated in a *motivational mindset model* (MMM). The motivational mindsets refer to different combinations of motives for studying that predispose students to reactions on their studies and study environment (Hudig et al., 2020). The four types of mindset in the MMM include the high-impact mindset, low-impact mindset, social-impact mindset, and self-impact mindset. Students with a high-impact mindset possess a strong drive on all aspects of their lives: earning high grades, having a rich social life, self-actualization, and a purposeful career. These students are future-oriented and hard-working and as they set high standards, they also have perfectionistic concerns. On the contrary, students with a low-impact mindset seem to have a shallow perspective on their future. These students have a passive attitude toward their studies, while they may be actively engaged in their social lives. Students with a social-impact mindset focus largely on personal growth and making a positive impact through their university study. Accordingly, these students wish to play a meaningful and instrumental role in society. They follow their interests and have idealistic concerns. Social-impact mindset students tend to show an open-minded attitude during study work. Finally, students with a self-impact mindset are particularly driven to achieve personal and financial success. They view their university studies as the “doorway” to this success. Self-impact mindset students are mostly money- and career-oriented.

Considering the potential value of the novel MMM for practitioners and for future research, Hudig et al. (2020) also developed the *mindset classification tool* (MCT). Rather than needing to always conduct a complex and demanding cluster analysis, this classification instrument enables individuals (e.g., researchers, study advisors, or teachers) to consistently classify students of any group size and in any setting into one of the motivational mindsets (Hudig et al., 2020). In their study, Hudig et al. (2020) conducted rigorous procedures to establish the internal validity of the MMM and MCT. An assessment of the motivational mindsets in relation to other measures, referring to the predictive or external validity (Bacher, 2002; Chittum and Jones, 2017), is however still lacking. Also, while the development of the MCT was done systematically, the actual usefulness of the instrument would benefit from a post-validation comparing the motivational mindsets in relevant student qualities. The MMM and the MCT have been developed with two goals in mind: (a) to better explain study success and student wellbeing, and (b) to understand why educational interventions are effective for some students and not so much for others (Hudig et al., 2020). If we find evidence for the predictive validity of the motivational mindsets in important aspects of study success and wellbeing, then this gives further indications that the motivational mindsets are indeed useful to reach these goals. The current paper therefore serves as a follow-up study aimed at validating the MMM and the newly developed instrument. To this end, sense of purpose and study engagement were used as the external variables and predictors of study success and student wellbeing.

### Sense of Purpose and Study Engagement

Purpose pertains to having a set of goals and a sense of direction for one’s life and is one of the central dimensions of mental wellbeing (Ryff, 1989; Dahl et al., 2020). It has also shown to be important for several other markers of wellbeing in adolescents and young adults (Bronk et al., 2009; Burrow et al., 2010; Burrow and Hill, 2011). Moreover, as students with purpose are more goal directed and possess a positive belief about dealing with potential obstacles, they are likely to perform better at school (Pizzolato et al., 2011). Purpose is therefore considered as a key developmental asset for students starting their academic program (Bronk, 2014).

Study engagement is a positive, fulfilling study-related state of mind comprising energy, dedication, and absorption (Schaufeli et al., 2002; Salanova et al., 2010; Salmela-Aro and Upadaya, 2012). Energy refers to high levels of vigor and a positive approach to studying. Dedication is characterized by a positive, cognitive attitude toward studying in general, a perception of studying as meaningful, and experiencing a sense of significance, enthusiasm, challenge, and inspiration. Finally, absorption is characterized by feelings of competence and being fully concentrated and happily engrossed in one’s studying so that time passes quickly. Study engagement is a central indicator of academic wellbeing and has extensively shown to be a strong promotor of academic performance (Schaufeli et al., 2002; Salanova et al., 2010; Tuominen-Soini et al., 2012; Ayala and Manzano, 2018; Widlund et al., 2018). Students who are engaged feel dedicated toward their studies and invest their time and energy into learning activities. Importantly, the level of study engagement at the start of university is considered especially crucial as it spills over into the first year and the rest of the study career (Ketonen et al., 2019). Both purpose and engagement are regarded developmental assets as they are facilitators of positive youth development (Benson et al., 2006).

### Present Study

The MMM is a translation of multiple, interacting motives for studying into a functioning and operating reality. The objectives of the present study are threefold and they aim to assess how well this translation or operationalization was done. Each of the three procedures are somehow related to construct validity. Construct validity speaks to the overarching quality of any operationalization (Trochim, 2006). The validity procedures aim to deepen our understanding of the motivational mindset profiles and establish the measurement quality of the MCT. To this end, we explore theoretically expected patterns as well as patterns that provide new insights relevant for future research.

The first objective is to assess the external or predictive validity of the MMM. To test this validity, the four mindset profiles should be associated with external variables that are theoretically and empirically related (Bacher, 2002; Chittum and Jones, 2017). In the next section, we lay out how purpose and study engagement relate to the motivational mindsets. If patterns in the results align with our expectations, this then provides evidence for predictive validity of the MMM. *Research Question 1* entails: “To what extent are there differences between the motivational mindsets in levels of (a) purpose and (b) study engagement?”

For the second objective, we investigate the relationship between sense of purpose and study engagement. Research suggests that there is a positive association between sense of purpose and study engagement. Purpose has been linked with academic engagement in a high-school setting before (Hill et al., 2018). This is, however, to the best of our knowledge the first study that explicitly connects these two core components of positive youth development in a university context. We first assess the purpose-engagement relationship in the overall sample. To provide evidence for the validity of the MMM and MCT, we expect this overall pattern to be similar across the four mindset profiles. *Research Question 2* is: “To what extent is sense of purpose related to study engagement, and does this relationship differ by motivational mindset?”

The third objective is an exploration into gender and ethnicity. One of the main goals of the approach within the MMM is to better understand differences in study success. Gender and ethnic background characteristics have been studied extensively in relation to study success. As stated earlier, purpose and engagement are predictors of study success and research on purpose and study engagement has shown that the gender and ethnic subgroups tend to vary in these student qualities. The present study therefore explores gender and ethnic differences in purpose and engagement among the four types of motivational mindsets. This exploration results in a kind of control exercise, which provides more depth to the validation of the motivational mindsets. Although we cannot expect certain patterns to emerge beforehand, if we observe meaningful patterns this then adds validity to the operationalization of the MMM. *Research Question 3* is: “Are there (a) gender and (b) ethnic differences in sense of purpose and study engagement, and do these differences vary between motivational mindsets?”

In light of the exploratory nature of the present study and the novelty of the MMM, we cannot yet postulate and test formal hypotheses. We therefore draft expectations about possible differences, after which the results may generate hypotheses to be tested in future research.

### Motivational Mindset Differences in Sense of Purpose

Students with a high-impact mindset have high intentions to accomplish personally meaningful, long-term aims through their studies, which include self-development and a strong desire to contribute to a better world (Hudig et al., 2020). Similarly, students with a social-impact mindset aim to pursue personal growth and wish to contribute to the lives of others through their university studies. Bronk and Finch (2010) showed that young people with long-term, other-oriented aims were more likely to be searching for a purpose and to have identified a purpose. In addition, research has shown that students with both intrinsic self-oriented and self-transcendent motives for their future career goals experienced greater levels of purpose (Yeager et al., 2012). High-impact mindset students have a relatively higher level of extrinsic motives and are more focused on a well-paid job compared to social-impact mindset students (Hudig et al., 2020). Such extrinsic motives have demonstrated a negative influence on how personally meaningful students regard their schoolwork (Yeager et al., 2014). Research has also shown that more motivation is not always better; in fact, the quality of the motivation matters more than the quantity (Vansteenkiste et al., 2009). Thus, one could expect a slightly stronger sense of purpose in social-impact mindset students compared to high-impact mindset students.

Students with a self-impact mindset are mostly self-oriented and focused on their personal success, while high-impact mindset and social-impact mindset have endorsed self-transcendent reasons for studying. As a prosocial orientation has shown to be a unique predictor of purpose (Hill et al., 2010), we expect that both the high-impact mindset and social-impact mindset students have a stronger sense of purpose than the self-impact mindset. Subsequently, we can expect that the self-impact mindset students feel a stronger sense of purpose than the low-impact mindset students. Low-impact mindset students have no real aspirations for growth nor to contribute to society (Hudig et al., 2020). Although self-impact students tend to be self-centered, their intention to accomplish personally meaningful goals could still produce feelings of purpose. Low-impact mindset students are directionless, while self-impact mindset students may have a sense of self-directedness. Based on the previous, we expect that the four mindsets gradually differ from each other with regard to their sense of purpose, in a way that social-impact mindset students have the strongest sense of purpose, then the high-impact mindset, then the self-impact mindset, and lastly, low-impact mindset students.

### Motivational Mindset Differences in Study Engagement

Study engagement is a committed and study-related state of mind comprising energy, dedication, and absorption (Upadyaya and Salmela-Aro, 2013). High-impact mindset students are future-oriented and hard-working, which signals a solid amount of dedication to their studies (Hudig et al., 2020). Also, as these students have high standards, they may often feel immersed in their study work. Having a high-impact mindset at the start of university should drive these students to invest time and effort into learning activities, although their excessive striving could result in exhaustion further into the academic program (Salmela-Aro and Read, 2017). Social-impact mindset students study primarily out of personal interest and a wish to help others through their studies (Hudig et al., 2020). This type of drive is illustrated by a sense of enthusiasm and a curiosity for study work, with curiosity being a force for daily levels of study engagement (Garrosa et al., 2017). The interest and enjoyment in learning of social-impact mindset students also elicit the perceived value of their study tasks and activities. This, in turn, likely bolsters their commitment to the study program (Wigfield and Eccles, 2000; Eccles, 2009). Since the high-impact mindset and social-impact mindset students both endorse self-transcendent reasons for studying, we suggest that this prosocial motivation leads to higher levels of dedication to learning activities. Students with a high-impact mindset go to university not only for intrinsic reasons, but also for career and financial success (Hudig et al., 2020). Due to the interaction of strong reasons on all dimensions, one could expect, although the disparity should be small, that high-impact mindset students have higher engagement levels than social-impact mindset students.

Self-impact mindset students go to university primarily for financial success and this kind of motivation has been negatively linked to academic engagement (King et al., 2017; King, 2018). Their primary focus on having a financially successful career may, however, generate the willingness to invest energy into learning activities. Moreover, self-impact mindset students draw their self-worth from their studies and feel proud to be at university. Although such extrinsic motives tend to create short-term persistence and seem detrimental for academic motivation in the long run (Vansteenkiste et al., 2006), these feelings could enhance students’ commitment to studying at the beginning of their studies. Students with a low-impact mindset, on the contrary, seem completely indifferent to learning (Hudig et al., 2020). As they find tasks and activities related to their studies uninteresting, they will only invest the time and effort into their study work when they absolutely need to (Symonds et al., 2019). Despite valuing their social lives, these students have not considered the value and meaning of their study program. Hence, we predict that they lack a sense of psychological engagement toward studying relative to the other mindsets.

Given the expected association between sense of purpose and study engagement, we regard purpose as a confounder when testing the motivational mindset differences in study engagement. Based on our lines of reasoning, we therefore expect that, controlling for sense of purpose, the four mindsets gradually differ from each other with respect to study engagement, in a way that high-impact mindset students have the highest level of study engagement, then the social-impact mindset, subsequently the self-impact mindset, and finally low-impact mindset students.

### Relationship Between Purpose and Study Engagement Across Mindsets

Exploring and identifying purpose entails higher-level cognitive processing (McKnight and Kashdan, 2009) and this same mechanism might help students understand the value and relevance of their studies, increasing engagement levels. Indeed, research has demonstrated that a sense of purpose increases the sense that schoolwork is meaningful (Yeager and Bundick, 2009). This, in turn, is likely to enhance students’ feelings of engagement at the start of the academic year (Vansteenkiste et al., 2018). Entering university is a challenging period for students (Trautwein and Bosse, 2017). Since purpose serves as a buffer in challenging times (Hill et al., 2016), students with purpose may utilize their psychological flexibility to engage in learning activities despite facing difficulties and distractions. Based on the previous, we expect that students’ sense of purpose is positively related to their study engagement. Since we expect a generic relationship between sense of purpose and study engagement, the relationship should not differ between the four motivational mindsets.

### Gender and Ethnic Differences

Educational researchers have been widely interested in gender and ethnicity to assess variation in study success. Research on study success often reports that male students and ethnic minority students perform less well and are more likely to drop out (Schippers et al., 2015). So far, several interpretations on different levels have attempted to explain these differences in study success between the gender and ethnic subgroups (e.g., Cotton et al., 2016). Yet it remains ambiguous what exactly causes this gender and ethnicity gap. A relatively small body of research has identified gender and ethnic differences in sense of purpose and in study engagement. Results showed that female students tend to have a stronger sense of purpose and higher engagement toward their studies compared to male students (Upadyaya and Salmela-Aro, 2015; Xi et al., 2018). Similarly, ethnic minority students have shown to possess a stronger sense of purpose and higher level of study engagement than majority students (Martinez and Dukes, 1997; Wang et al., 2011). Notably, these studies were conducted with African Americans as the minority group.

Regardless of these differences between gender and ethnic subgroups, the background characteristics of students in and of itself do not provide a substantive explanation for differences in study success, nor for the differences in purpose and study engagement. Hence, we aimed to investigate the role of gender and ethnicity in the MMM. There is insufficient empirical guidance to state concrete expectations about gender and ethnic subgroup differences among the four types of mindset. We first observe in what way the gender and ethnic subgroups are distributed over the varying mindsets. Next, we assess whether the gender and ethnic subgroups overall differ in purpose and study engagement. And finally, we explore to what extent subgroup differences in these student qualities occur across the four motivational mindsets.

## Materials and Methods

### Sample and Procedure

The total sample involved 1,011 commencing, first-year university students from a business school in Netherlands. All participants were enrolled in the Bachelor program business administration and came from the same cohort (i.e., 2018–2019). As a questionnaire was used (see below), 905 students responded (89.52%) and 852 (84.27%) students completed the questionnaire. From these 852 participants, 104 students did not provide consent to process their data and we removed the data of those students from the dataset. In addition, while developing and validating the MCT, several students were detected with a combination of motives that did not fit well with any of the four motivational mindsets. These students were then withdrawn from the mindset profiles and classified into the residual group (for details, see Hudig et al., 2020). For the purpose of the present study, the residual group (*n*=86) was not further analyzed and these students were removed from the sample. As a result, the final sample consisted of 662 students. In terms of gender, the sample comprised 442 men (66.8%) and 220 women (33.2%). Their ages ranged from 17 to 30years (M=18.50; SD=1.15) and 11.3% of the total sample were non-western ethnic minority students.^1^

The data of the current study were collected after approval from the Internal Review Board of the research school. The data processing procedures were also assessed through a Data Protection Impact Assessment and adhered to all General Data Protection Rules guidelines. Data collection consisted of an online survey and drawing on the academic records of the university. The self-report questionnaire including items on motives for studying, purpose, and study engagement was sent to students *via* Qualtrics. Students filled in these questionnaires at home in the first month of their first semester at university. Prior to starting the questionnaire, students were provided with a consent form in which they had to explicitly approve their participation in the research. The purpose of the research was explained to them and it was underscored that their participation was voluntary. They also had the possibility to withdraw their data from the research at any time and it was emphasized that the data were treated confidentially. Data on gender, ethnicity, and age were collected at the end of the academic year by making use of the academic records.

### Measures

#### Motivational Mindsets

The motivational mindsets were established based on three dimensions of the Dutch *study motives scale* (SMC; Hudig et al., 2020). Each dimension comprised three items: (1) Self-transcendent motives (*α*=0.65 in Hudig et al., 2020): “I want to learn things that will help me make a positive impact on the world,” “I want to gain skills that I can use in a job that help others,” and “I want to become an educated citizen that can contribute to society.”(2) Self-oriented, intrinsic motives (*α*=0.68 in Hudig et al., 2020): “I want to expand my knowledge of the world,” “I want to become an independent thinker,” and “I want to learn more about my interests.” (3) Extrinsic motives (*α*=0.62 in Hudig et al., 2020): “I want to get a good job,” “I want to earn more money,” and “I want to have fun and make new friends.” All items were rated on a Likert scale ranging from 1 (*totally disagree*) to 5 (*totally agree*).

To classify students into the motivational mindset profiles based on their scores on the SMC, the MCT was employed. First, frequency tables for each motivational dimension were computed (without outliers ±3 SD above and below the mean) and the difference between the highest and lowest score on each study motives subscale was calculated. This range difference was then divided by three to establish three criteria values for each subscale indicating a low-, middle-, or high-level score. Then, per student, each individual numerical value on the three motivational dimensions were transformed into three score levels accordingly. Finally, outliers were reincluded and students were allocated to one of the mindset groups based on their pattern of these three levels (for more details on the MCT, see Hudig et al., 2020).

#### Sense of Purpose

Sense of purpose was measured with the Purpose in Life subscale from the Scales of Psychological Well-Being (Ryff, 1989). Several versions of the PIL subscale exist varying from 20 to 3 items. Following Ryff’s personal recommendation from the Abbott et al. (2010) study, we adopted the 7-item version and translated this version into Dutch. Seven items (e.g., I enjoy making plans for the future and working to make them a reality) were rated on a Likert scale ranging from 1 (*totally disagree*) to 5 (*totally agree*). Prior to analyzing, items with negative content were reverse scored so that high values indicated greater sense of purpose. Previous research has recorded the validity of the Purpose in Life subscale (Ryff, 1989; Keyes and Ryff, 1995) and a mean Cronbach’s alpha of 0.74 for the 7-item version (Crouch et al., 2017). After we had collected the data, we discovered a Dutch version of the PIL scale that was already validated in a university student sample (van Dierendonck, 2004). To post-validate our translation, two independent raters compared our translated items with the translation of van Dierendonck (2004). Each item was rated on a scale from 0 to 100% to indicate the equivalence in meaning. From these ratings, the total average per rater was acceptable (rater 1=73.57%; rater 2=86.14%; and overall=79.86%). One of the items (i.e., I used to set goals for myself, but that now seems a waste of time) was rated relatively low (particularly by rater 2). Yet we felt this was more attributable to the sentence construction than the substantive meaning of the item. Hence, based on these ratings, we regarded our translation adequately similar to the validated translation of van Dierendonck (2004).

#### Study Engagement

Study engagement was measured with the Utrecht Work Engagement Scale for students (UWES-S; Schaufeli et al., 2002). The shortened 9-item Dutch version of the instrument was used of which the internal reliability and construct validity have been well established (Schaufeli and Bakker, 2003). Schaufeli and Bakker (2003) showed that the total 9-item scale had a Cronbach’s alpha of 0.84 and the three subscales (energy, dedication, and absorption) with each three items had internal consistencies of 0.73, 0.76, and 0.70, respectively. Example items are: “When I study, I feel like I am bursting with energy” (energy), “I find my study useful and meaningful” (dedication), and “Time flies when I’m studying” (absorption). All items were rated on a Likert scale ranging from 1 (*totally disagree*) to 5 (*totally agree*). Both the one-factor and three-factor structure of the UWES-S have been supported (Schaufeli and Bakker, 2003; Salmela-Aro and Upadaya, 2012). As the three dimensions are very closely related and have shown to not differentiate the students (Schaufeli et al., 2006; Tuominen-Soini and Salmela-Aro, 2014), engagement was used in this research as a single dimension indicating overall study engagement.

### Analyses

Before exploring the research questions, missing data and outliers were examined (Hair et al., 2013). Subsequently, the internal reliability of the measures was assessed. After screening the data and before examining every hypothesis, the relevant assumptions were considered to prevent potential bias in the statistical tests (Field, 2013). To explore the motivational mindset differences in sense of purpose (RQ1a), we conducted an ANOVA and *post-hoc* comparisons with motivational mindsets (between-subjects) as the independent variable (IV) and sense of purpose as the dependent variable (DV). To examine the motivational mindset differences in study engagement when controlling for sense of purpose (RQ1b), we conducted an ANCOVA and *post-hoc* comparisons with motivational mindsets (between-subjects) as the IV, sense of purpose as the covariate, and study engagement as the DV. To explore the relationship between sense of purpose and study engagement (RQ2), a correlational analysis was conducted in the full sample and within each motivational mindset. To explore differences in levels of purpose and study engagement between gender (RQ3a) and ethnic (RQ3b) subgroups across motivational mindsets, we performed a series of univariate ANOVAs and *post-hoc* comparisons.^2^ The first two ANOVAs included gender (between-subjects) as IV and purpose and study engagement as the DV. Subsequently, four identical ANOVAs were performed within each motivational mindset. Furthermore, two ANOVAs were performed which included ethnicity (between-subjects) as IV and purpose and study engagement as the DV. Similarly, four identical ANOVAs were performed within the four motivational mindsets. Each *post-hoc* test was conducted with a Bonferroni correction to avoid false significant results (Cabin and Mitchell, 2000). The *post-hoc* tests included six mindset comparisons: (1) high-impact vs. low-impact, (2) social-impact vs. low-impact, (3) self-impact vs. low-impact, (4) social-impact vs. high-impact, (5) social-impact vs. self-impact, and (6) high-impact vs. self-impact. Effect sizes were calculated using Cohen’s *d* (0.20/0.50/0.80=small/medium/large effect size; Cohen, 1992; Horn, 2007) and all statistical analyses were executed using SPSS version 25.

## Results

### Preliminary Analysis

We first examined missing data. On the item-level, we identified 8 missing values on the PIL scale (0.17%) and 5 missing values on the UWES-S (0.08%). As the amount of missings was very small and each missing value concerned a separate case, we diagnosed the missings completely at random, and we ignored the missing data (Hair et al., 2013). After screening the data, no outliers or extreme cases were removed. Next, we assessed the internal consistency of the measures. Table 1 displays the descriptive statistics, Cronbach’s alpha’s, and intercorrelations among the variables of this study. The SMC noted a Cronbach’s alpha of 0.67, 0.68, and 0.65 for the self-transcendent, self-oriented, and extrinsic dimensions, respectively. The PIL scale showed a Cronbach’s alpha of 0.72 and the UWES-S revealed a Cronbach’s alpha of 0.81. Although some of the reliability coefficients were low by most standards (<0.8), they were all acceptable (Hair et al., 2013; Taber, 2018). The mindset classification produced the following distribution of students: 193 high-impact mindset students, 94 low-impact mindset students, 186 social-impact mindset students, and 189 self-impact mindset students (see also Table 2).

**Table 1:** Descriptive statistics, Cronbach’s alpha coefficients, and intercorrelations.

	No. of items	M	SD	*α*	*r*				
					1	2	3	4	5
(1) Self-transcendent motives	3	3.96	0.64	0.67	–				
(2) Self-oriented, intrinsic motives	3	4.23	0.60	0.68	0.45^**^	–			
(3) Extrinsic motives	3	4.29	0.64	0.64	0.15^**^	0.28^**^	–		
(4) Sense of purpose	7	3.89	0.53	0.72	0.25^**^	0.20^**^	−0.01	–	
(5) Study engagement	9	3.50	0.52	0.81	0.40^**^	0.37^**^	0.01	0.47^**^	–

*n = 662*.

** *p < 0.001*

**Table 2:** Student distribution by sample, motivational mindsets, gender, and ethnic subgroups.

		Gender		Ethnicity	
		Female	Male	Minority	Majority
	*n*	*n*	*n*	*n*	*n*
Full sample	662 (100%)	220 (33.2%)	442 (66.8%)	75 (11.3%)	540 (81.6%)
High-impact mindset	193 (29.2%)	80 (41.5%)	113 (58.5%)	25 (13%)	154 (79.8%)
Low-impact mindset	94 (14.2%)	17 (18.1%)	77 (81.9%)	10 (10.6%)	79 (84%)
Social-impact mindset	186 (28.1%)	77 (41.4%)	109 (58.6%)	22 (11.8%)	151 (81.2%)
Self-impact mindset	189 (28.5%)	46 (24.3%)	143 (75.7%)	18 (9.5%)	156 (82.5%)

*Ethnicity does not sum to 100% because Western minority students (n=47) were excluded from comparing analyses*.

### Motivational Mindset Differences in Purpose

**Figure 1:**
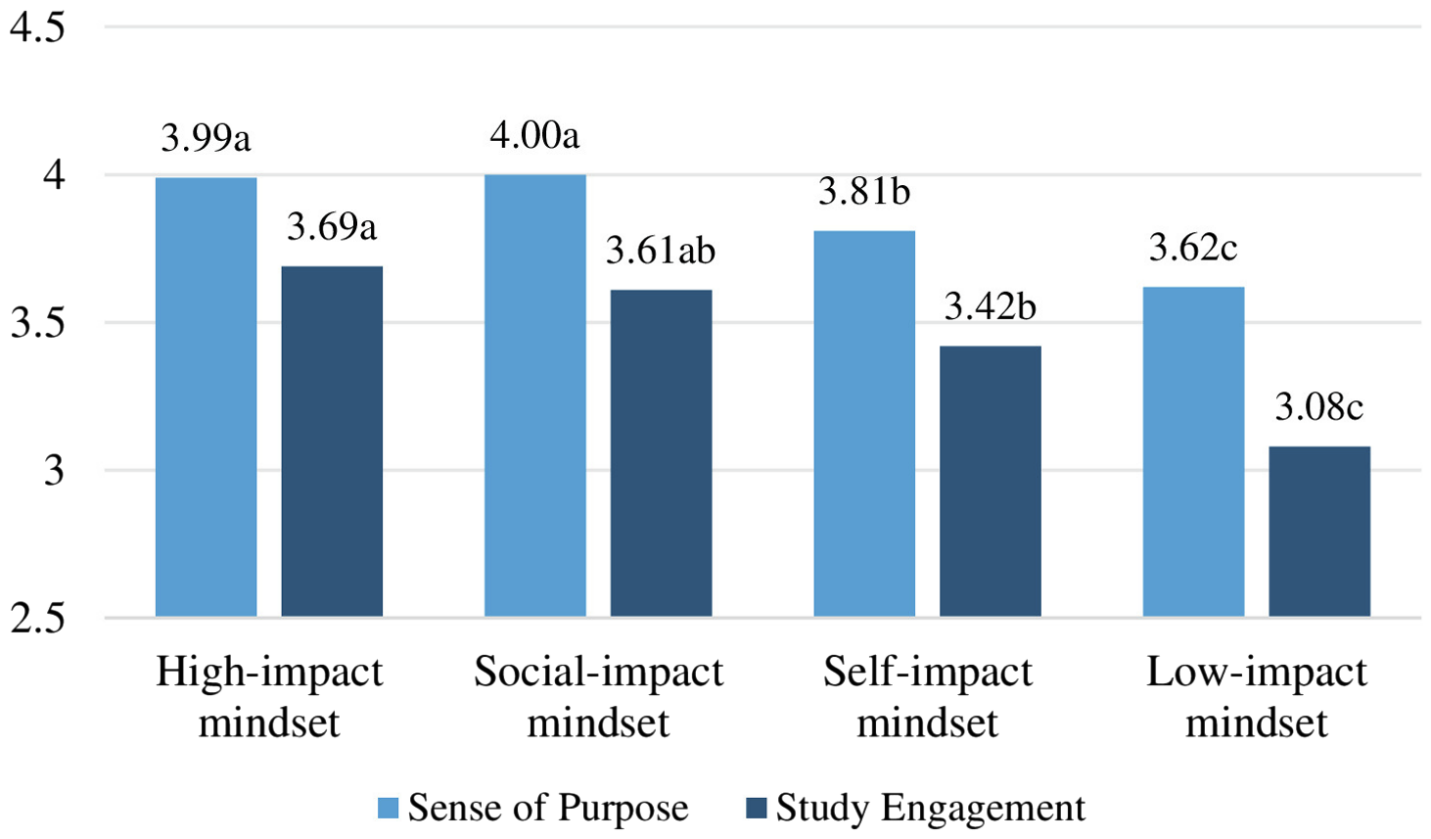
Student mean levels in sense of purpose and study engagement. (Different letters denote significant differences *between* motivational mindsets).

To explore motivational mindset differences in sense of purpose, we conducted an ANOVA and *post-hoc* tests. Prior to performing the analysis, we considered the relevant assumptions. To rectify for unequal variances between the mindset groups, we used a corrected version of the *F* ratio and Games-Howell corrected *post-hoc* (Horn, 2007). The motivational mindsets showed to be significantly related to sense of purpose, Welch’s adjusted *F* ratio (3,304.64)=13.88, *p*<0.001, *est. ω*^2^=0.06. *Post-hoc* tests with Games-Howell corrected values of *p* revealed the following for the six mindset comparisons. Mean levels of purpose and significant differences are displayed in Figure 1 and Table 3.

**Table 3:** Mean-level analysis sense of purpose.

				Gender						Ethnicity					
				Female			Male			Minority			Majority		
	*n*	M	SD	*n*	M	SD	*n*	M	SD	*n*	M	SD	*n*	M	SD
Full sample	662	3.89	0.53	220	3.98a	0.49	442	3.84b	0.54	75	3.94	0.54	540	3.87	0.52
High-impact mindset	193	3.99a	0.53	80	4.03	0.50	113	3.94	0.55	25	4.03	0.49	154	3.96	0.54
Low-impact mindset	94	3.62c	0.57	17	3.58	0.63	77	3.63	0.56	10	3.69	0.77	79	3.62	0.55
Social-impact mindset	186	4.00a	0.45	77	4.04	0.46	109	3.98	0.44	22	4.11	0.49	151	3.97	0.44
Self-impact mindset	189	3.81b	0.52	46	3.97a	0.42	143	3.76b	0.54	18	3.75	0.42	156	3.83	0.50

*Letters denote post-hoc comparisons – different letters indicate significant different means between mindsets in the full sample, between subgroups in the full sample, or between subgroups within mindset*.

The high-impact mindset compared to the low-impact mindset showed significantly higher levels of purpose (*comparison 1*) with mean difference, M=0.36, CI (0.17, 0.54), *t*(304.64)=5.09, *p*<0.001, *d*=0.66. The social-impact mindset showed significantly higher levels of purpose compared to the low-impact mindset (*comparison 2*) with mean difference, M=0.38, 95% CI (0.21, 0.56), *t*(304.64)=5.70, *p*<0.001, *d*=0.74. The self-impact mindset compared to the low-impact mindset showed significantly higher levels of purpose (*comparison 3*) with mean difference, M=0.19, 95% CI (0.01, 0.38), *t*(304.64)=2.79, *p*=0.030, *d*=0.35. The social-impact mindset compared to the high-impact mindset did not show significantly higher levels of purpose (*comparison 4*) with mean difference, M=0.03, 95% CI (−0.10, 0.16), *t*(304.64)=0.54, *p*=0.948. The social-impact mindset compared to the self-impact mindset showed significantly higher levels of purpose (*comparison 5*) with mean difference, M=0.19, 95% CI (0.06, 0.32), *t*(304.64)=3.79, *p*=0.001, *d*=0.39. The high-impact mindset compared to the self-impact mindset (*comparison 6*) also showed significantly higher levels of purpose with mean difference, M=0.16, 95% CI (0.02, 0.30), *t*(304.64)=3.02, *p*=0.014, *d*=0.31. So, from the six comparisons that we made, five were in line with our expectations, ranging from medium-low to medium-large effect sizes. Only the social-impact mindset compared to the high-impact mindset did not show significantly higher levels of purpose.

### Motivational Mindset Differences in Study Engagement

To explore the motivational mindsets differences in study engagement when controlling for preexisting levels of purpose, we conducted an ANCOVA and *post-hoc* tests. The required assumptions were checked and confirmed. The analysis showed that the covariate, sense of purpose, was significantly related to study engagement, *F*(1,657)=136.48, *p*<0.001, partial *η*^2^=0.17. Moreover, the motivational mindsets were significantly related to study engagement after controlling for sense of purpose, *F*(3,657)=20.03, *p*<0.001, partial *η*^2^=0.10. Taken together, 27% of the variance in study engagement could be explained by students’ sense of purpose and the motivational mindsets. *Post-hoc* tests with Bonferroni-corrected values of *p* revealed the following for the six comparisons. Mean levels of study engagement and significant differences between mindsets are displayed in Figure 1 and Table 4.

**Table 4:** Mean-level analysis study engagement.

				Gender						Ethnicity					
				Female			Male			Minority			Majority		
	*n*	M	SD	*n*	M	SD	*n*	M	SD	*n*	M	SD	*n*	M	SD
Full sample	662	3.50	0.52	220	3.63a	0.51	442	3.44b	0.51	75	3.72a	0.47	540	3.47b	0.51
High-impact mindset	193	3.69a	0.48	80	3.79a	0.50	113	3.61b	0.45	25	3.86	0.40	154	3.65	0.49
Low-impact mindset	94	3.08d	0.52	17	3.02	0.60	77	3.09	0.51	10	3.29	0.55	79	3.06	0.52
Social-impact mindset	186	3.61ab	0.46	77	3.67	0.38	109	3.56	0.50	22	3.76	0.51	151	3.58	0.42
Self-impact mindset	189	3.42b	0.48	46	3.50	0.51	143	3.39	0.46	18	3.71a	0.37	156	3.39b	0.47

*Letters denote post-hoc comparisons – different letters indicate significant different means between mindsets in the full sample, between subgroups in the full sample, or between subgroups within mindset*.

The high-impact mindset compared to the low-impact mindset showed significantly higher levels of study engagement (*comparison 1*) with (estimated marginal) mean difference, M=0.47, CI (0.32, 0.62), *t*(657)=8.38, *p*<0.001, *d*=1.08. The social-impact mindset also showed significantly higher levels of study engagement compared to the low-impact mindset (*comparison 2*) with mean difference, M=0.38, 95% CI (0.23, 0.53), *t*(657)=6.61, *p*<0.001, *d*=0.87. The self-impact mindset compared to the low-impact mindset showed significantly higher levels of study engagement (*comparison 3*) with mean difference, M=0.26, 95% CI (0.12, 0.41), *t*(657)=4.78, *p*<0.001, *d*=0.60. The social-impact mindset compared to the high-impact mindset did not show significantly higher levels of study engagement (*comparison 4*) with mean difference, M=−0.09, 95% CI (−0.21, 0.03), *t*(657)=−2.02, *p*=0.252. The social-impact mindset compared to the self-impact mindset also did not show higher levels of study engagement (*comparison 5*) with mean difference, M=0.11, 95% CI (−0.01, 0.23), *t*(657)=2.53, *p*=0.073. Finally, the high-impact mindset compared to the self-impact mindset showed significantly higher levels of study engagement (*comparison 6*) with mean difference, M=0.21, 95% CI (0.09, 0.32), *t*(657)=4.75, *p*<0.001, *d*=0.49. Concluding, from the six comparisons that we made, four were in line with our expectations, showing medium to large effect sizes. Only the social-impact mindset compared to the high-impact mindset, and the social-impact mindset compared to the self-impact mindset did not show significantly different levels of study engagement.

### Relationship Between Purpose and Study Engagement Across Mindsets

Prior to performing the analyses, the relevant assumptions of normality and linearity were assessed and confirmed. To examine the relation between sense of purpose and study engagement, and whether similar patterns were noted for all motivational mindsets, we inspected the bivariate zero-order correlations. Overall, in the full sample, sense of purpose was positively correlated with study engagement *r* (662)=0.47, *p*<0.001. We then inspected the purpose-study engagement association for each mindset group. In the high-impact mindset, sense of purpose was positively correlated with study engagement, *r* (193)=0.37, *p*=0.001. In the low-impact mindset, the correlation between sense of purpose and study engagement was again positive, *r* (94)=0.51, *p*<0.001. In the social-impact mindset, sense of purpose was positively correlated with study engagement, *r* (186)=0.42, *p*<0.001. Similarly, the self-impact mindset demonstrated a positive correlation between sense of purpose and study engagement, *r* (189)=0.41, *p*<0.001. The strength of these correlation coefficients all ranged from medium to large (0.10/0.30/0.50=small/medium/large; Cohen, 1992). According to Fisher’s test of the difference between two independent correlations (Lowry, 2001), none of the associations were statistically different for the varying motivational mindsets. Based on these results, the positive relationship between purpose and study engagement was confirmed and the relationship demonstrated consistency across the four mindsets.

### Gender Differences Across Mindsets

Table 2 presents the distribution of students by motivational mindset in terms of gender. Relative to the mean gender distribution in the sample, female students were overrepresented in the high-impact mindset and social-impact mindset, while underrepresented in the self-impact mindset and low-impact mindset. For male students, the observation is vice versa.

To further explore gender differences in sense of purpose and study engagement, and whether these differences vary between motivational mindsets, we performed two separate ANOVAs (one ANOVA for sense of purpose and one ANOVA for study engagement) and Bonferroni-corrected *post-hoc* tests. We assessed these gender differences in the full sample and in each motivational mindset. Prior to the analysis, we considered and confirmed the relevant assumptions.

In the full sample, gender showed to have a main effect on sense of purpose, *F*(1,660)=11.75, *p*=0.001, partial *η*^2^=0.02. Female students reported higher levels of purpose than male students with mean difference, M=0.15, 95% CI (0.06, 0.23), *t*(660)=3.33, *p*=0.001, *d*=0.29. Similarly, gender demonstrated a main effect on study engagement in the full sample, *F*(1,660)=20.26, *p*<0.001, partial *η*^2^=0.03. Female students reported significantly higher levels of study engagement than male students with mean difference, M=0.19, 95% CI (0.11, 0.27), *t*(660)=4.52, *p*<0.001, *d*=0.37. Tables 3 and 4 show the average mean levels of purpose and study engagement, and significant gender differences within the full sample.

**Figure 2:**
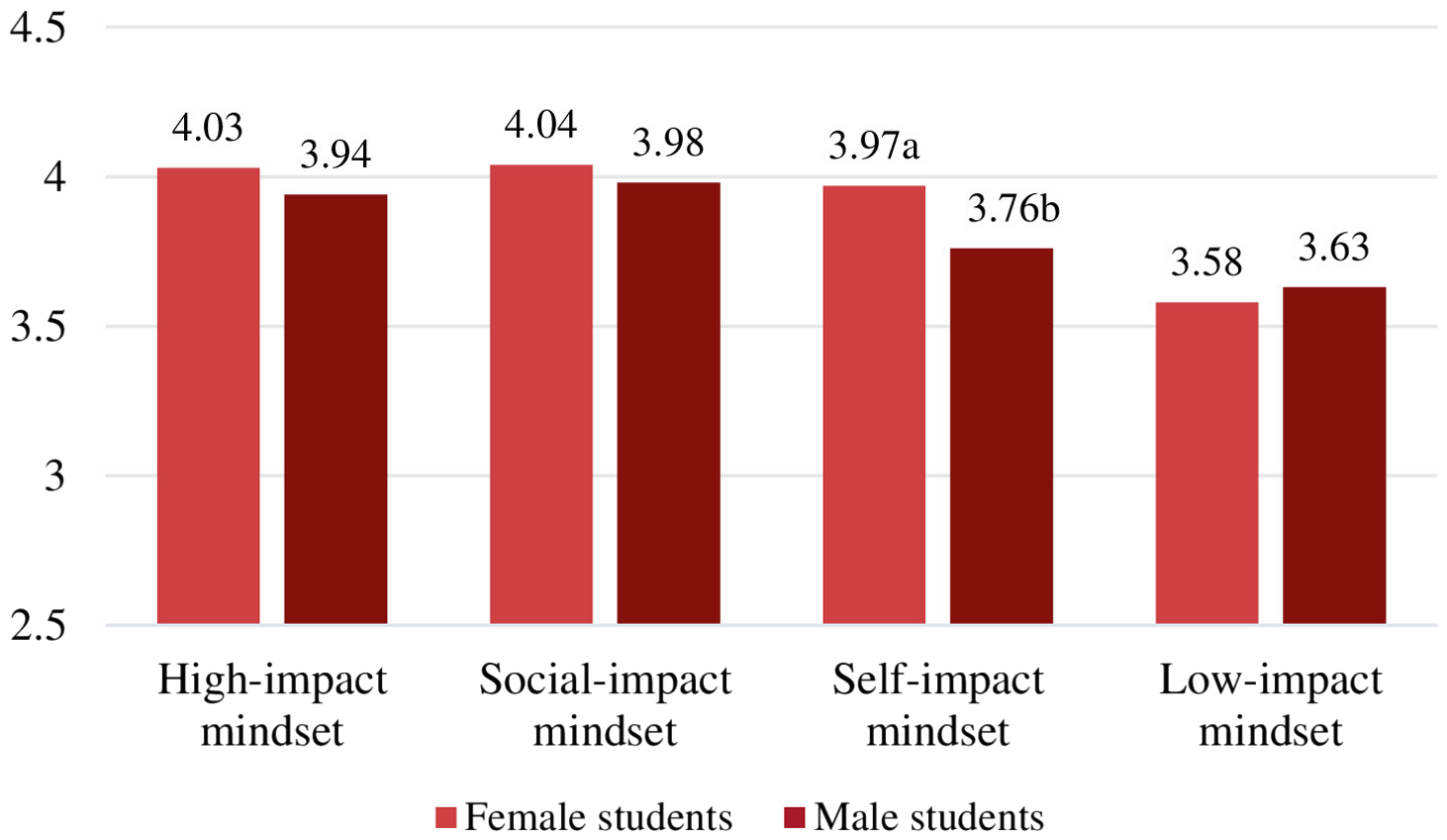
Gender group mean levels in sense of purpose. (Different letters denote significant differences between subgroups *within* mindset).

Then, we assessed the levels of purpose for the gender groups by motivational mindset. When inspecting levels of purpose for the gender groups within the motivational mindsets, only in the *self-impact mindset* did gender show to have a main effect on sense of purpose, *F*(1,187)=5.64, *p*=0.019, partial *η*^2^=0.03. Female students showed to have higher levels of purpose in this mindset than male students with mean difference, M=0.21, 95% CI (0.04, 0.38), *t*(187)=2.37, *p*=0.019, *d*=0.43. The mean levels of purpose and significant gender differences by each motivational mindset can be found in Table 3 and Figure 2.

**Figure 3:**
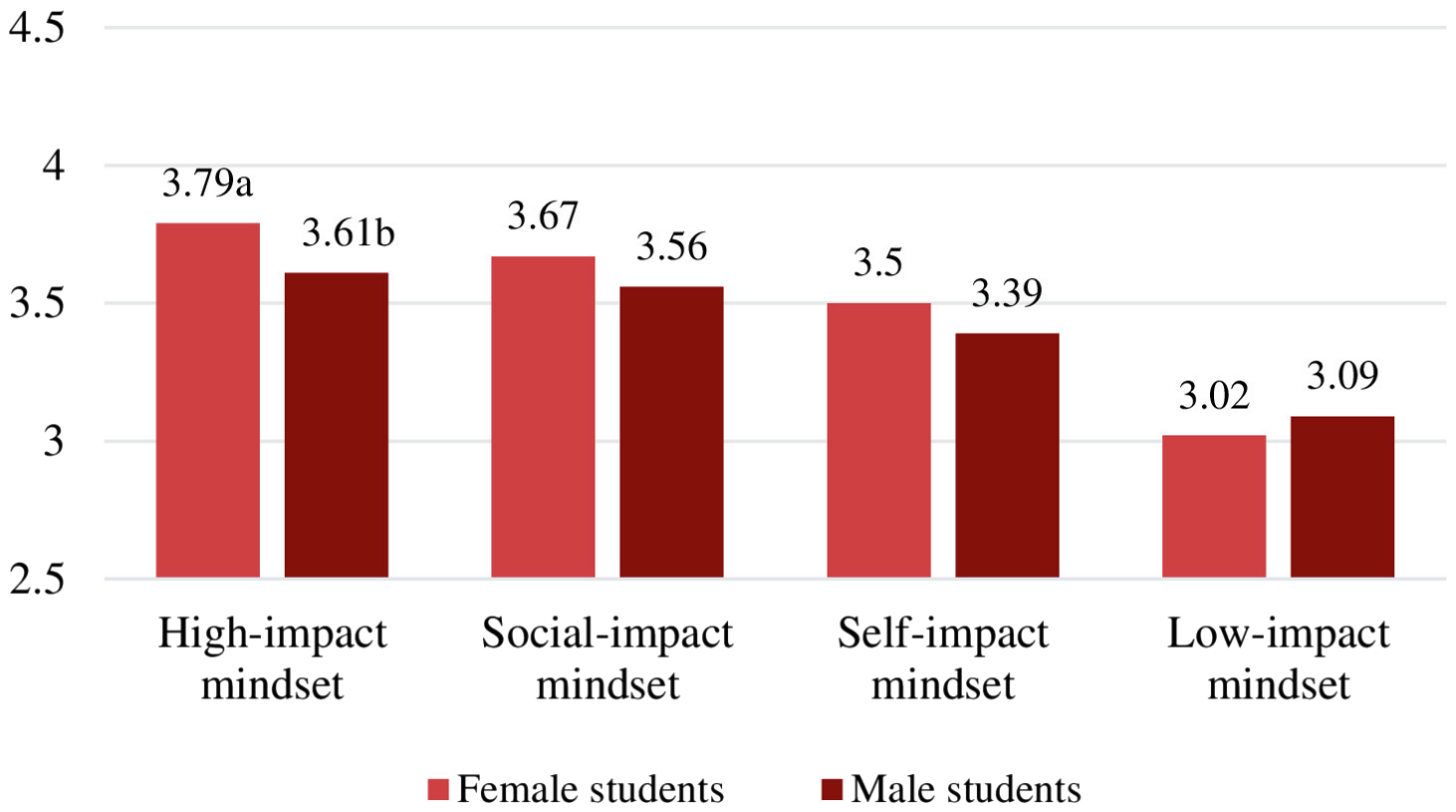
Gender group mean levels in study engagement. (Different letters denote significant differences between subgroups *within* mindset).

When inspecting levels of study engagement for the gender subgroups within the motivational mindsets, only in the *high-impact mindset* did gender show a significant main effect, *F*(1,191)=6,86, *p*=0.010, partial *η*^2^=0.04. Similar as in the overall sample, female students reported higher levels of study engagement than male students with mean difference, M=0.18, 95% CI (0.04, 0.32), *t*(191)=2.61, *p*=0.010, *d*=0.38. The mean levels of study engagement and significant gender differences by motivational mindset are shown in Table 4 and Figure 3.

### Ethnic Differences Across Mindsets

Table 2 presents the distribution of students by motivational mindset in terms of ethnicity. The distribution of minority and majority student across the four mindsets correspond closely with the mean ethnicity distribution in the sample.

To further explore ethnic differences in sense of purpose and study engagement, and whether these differences vary between motivational mindsets, we again performed two separate ANOVAs. The assumptions were met and in the full sample, ethnicity showed no main effect on sense of purpose, *F*(1,613)=1.13, *p*=0.288, partial *η*^2^=0.00. Conversely, ethnicity did show a main effect on study engagement, *F*(1,613)=16.37, *p*<0.001, partial *η*^2^=0.03. Ethnic minority students reported significantly higher levels of study engagement than majority students in the full sample with mean difference, M=0.25, 95% CI (0.13, 0.37), *t*(613)=4.05, *p*<0.001, *d*=0.51. Tables 3 and 4 show the average mean levels of purpose and study engagement in the full sample and significant ethnic subgroup differences.

**Figure 4:**
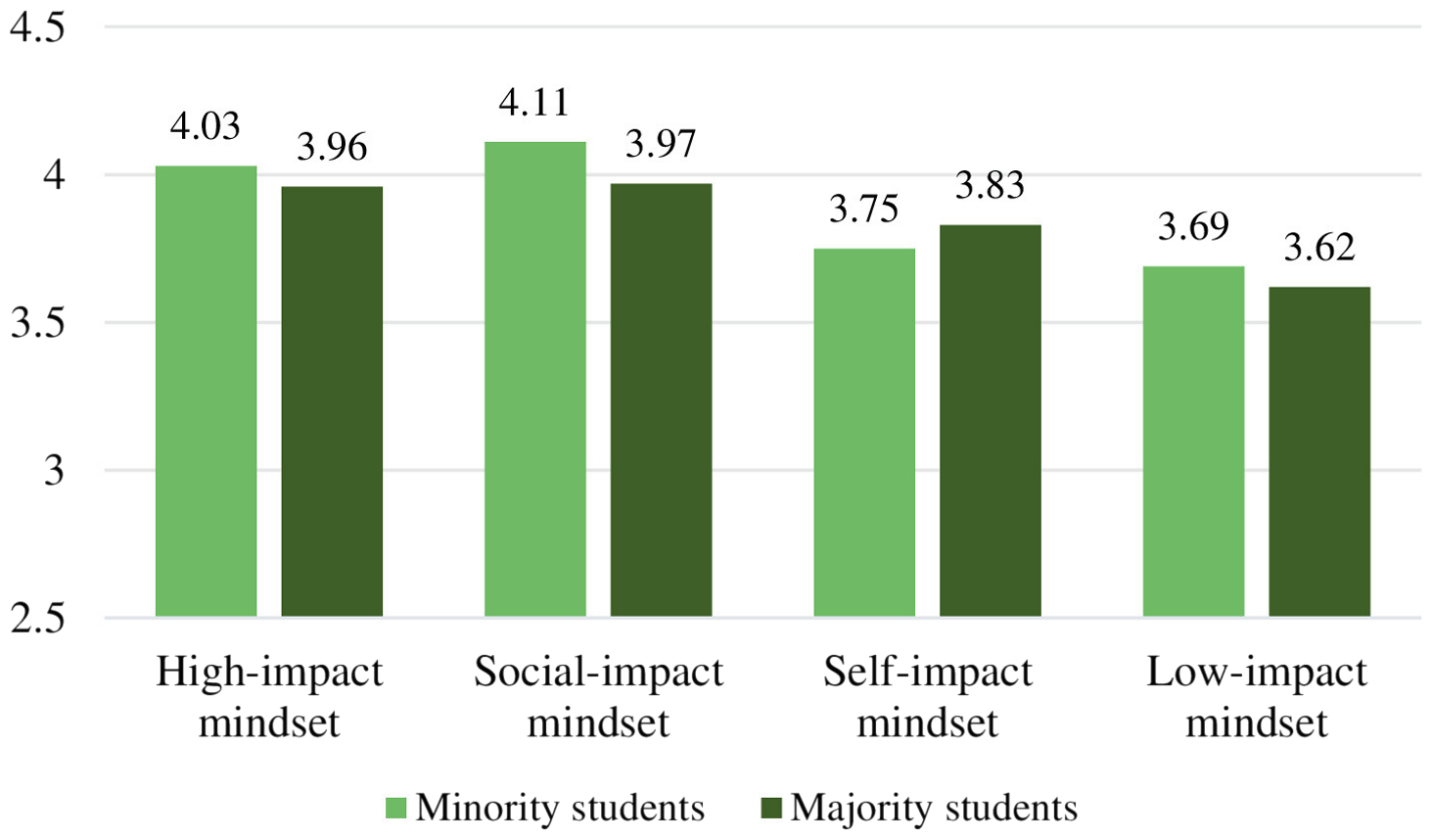
Ethnic group mean levels in sense of purpose.

When inspecting levels of purpose for the ethnic groups by motivational mindset, none of the mindsets revealed significant differences. The mean levels of purpose and non-significant ethnic subgroup differences in purpose are displayed in Table 3 and Figure 4.

**Figure 5:**
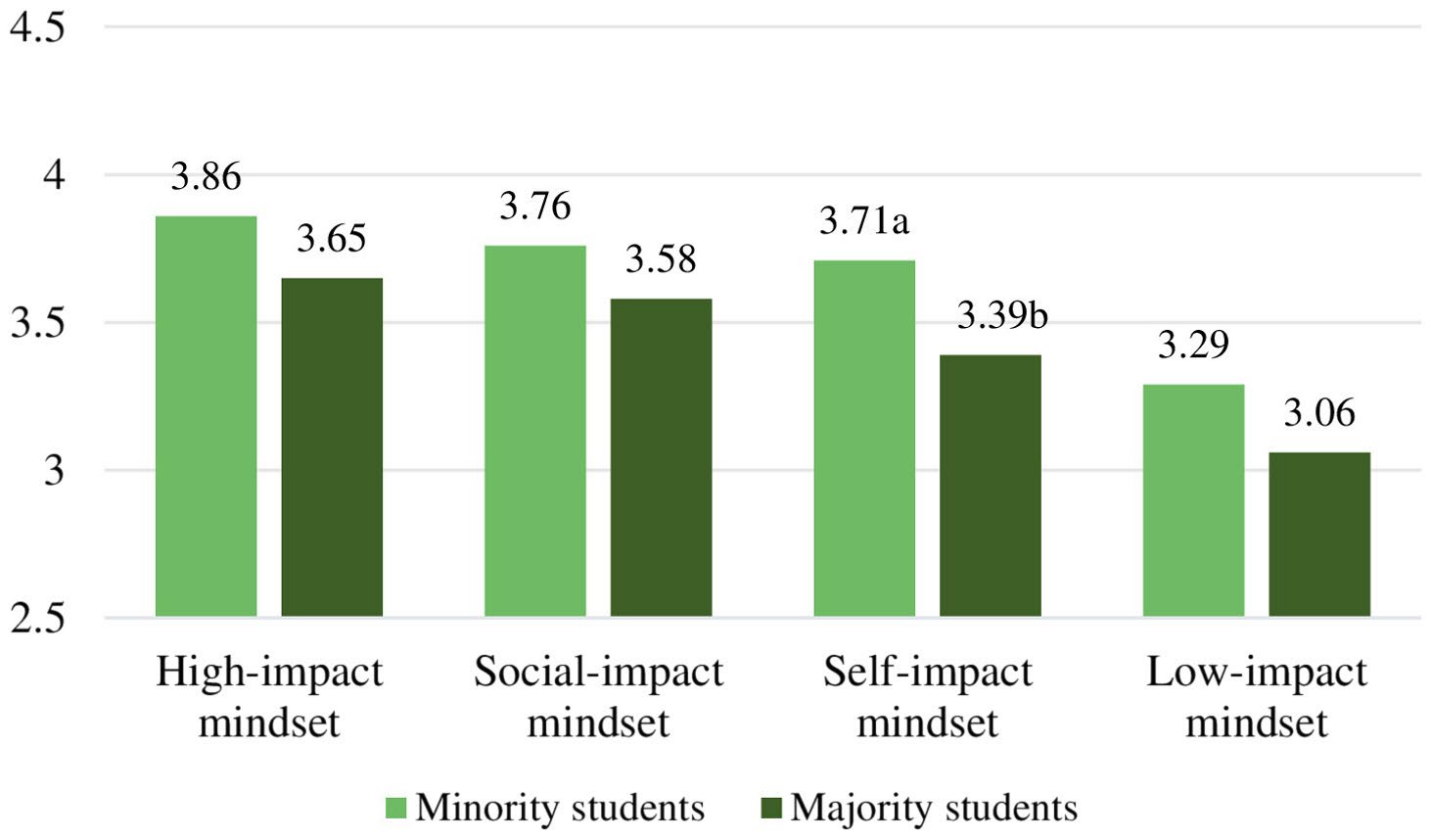
Ethnic group mean levels in study engagement. (Different letters denote significant differences between subgroups *within* mindset).

When inspecting levels of study engagement for the ethnic subgroups by motivational mindset, only in the *self-impact mindset* did ethnicity reveal to have a main effect on study engagement, *F*(1,186)=3.92, *p*=0.022, partial *η*^2^=0.04. The minority students showed higher levels of study engagement than the majority students with mean difference, M=0.32, 95% CI (0.04, 0.60), *t*(186)=2.74, *p*=0.020, *d*=0.76. The mean level of study engagement per mindset and significant ethnic subgroup differences is displayed in Table 4 and Figure 5.

## Discussion

This follow-up study aimed to validate the recently introduced MMM and the associated MCT. The MMM includes four motivational mindsets of which each mindset type consists of multiple, co-occurring motives for studying. This multidimensional perspective intends to provide a more realistic picture of the psychology of students in higher education (Hudig et al., 2020). Moreover, the interaction of motivational dimensions could potentially better explain differences in student performance and student wellbeing. Yet, considering the novelty of both the model and the instrument, a series of validation procedures was appropriate to further establish their value and usefulness. The first procedure was to externally validate the motivational mindsets by exploring their differences in two important predictors of study success and central dimensions of student wellbeing, namely, sense of purpose and study engagement. The results indicated that sense of purpose and study engagement differ across the four types of mindset with the low-impact mindset having the least optimal pattern of study engagement and sense of purpose. Second, as research has suggested a relationship between purpose and study engagement, we examined this association and subsequently, checked the similarity of the observation across the four mindsets. The positive relationship between sense of purpose and study engagement was confirmed and this relationship was consistent across mindsets. Finally, as gender and ethnicity have shown to be relevant in research on purpose, study engagement, and study success, we checked for gender and ethnic differences across the varying motivational mindsets. The results demonstrated that overall differences in purpose and study engagement between gender and ethnic subgroups emanated from one specific type of motivational mindset.

### Expansion of the Motivational Mindset Characteristics

The results showed that there are significant differences with respect to the different mindsets in relation to purpose and study engagement. First, students with a low-impact mindset reported substantially lower levels of purpose and study engagement than the other three mindsets. Given the characteristics of the low-impact mindset, this finding is not surprising. Low-impact mindset students have a shallow perspective of their future (Hudig et al., 2020). Although they go to university to enjoy their social lives, they study merely because it is expected of them and they have no intrinsic reasons for studying. Accordingly, they maintain a passive attitude to study work. As the results demonstrated that the low-impact mindset students reported a lack of direction and little experience of psychological engagement toward their studies, the findings validate and extend the description of this type of student mindset.

The pattern of results also showed that students with a social-impact mindset and high-impact mindset have a stronger sense of purpose than students with a self-impact mindset and low-impact mindset. This result is in line with studies affirming the value of self-transcendent motives to foster feelings of purpose (Yeager et al., 2014). The result also implies that students with self-transcendent reasons for studying have a stronger sense of purpose than students with just having self-focused sources of meaning. Researchers have emphasized to distinguish purpose from meaning and other long-term aims by its other-oriented focus (Damon et al., 2003; Bronk and Finch, 2010). Our finding seems to add more empirical evidence to that assertion.

Despite this student difference in purpose between the social-impact mindset and the self-impact mindset, they reported similar levels of study engagement. Self-impact mindset students study mainly for personal and financial success. This coincides with fewer feelings of purpose than studying primarily to be benefit for society as is the case among social-impact mindset students. Nonetheless, being highly self-focused can generate equal amounts of dedication and willingness to invest energy into studying. At least at the beginning of the academic program. One must consider that extrinsic, self-oriented reasons have shown to debilitate academic motivation over time (Vansteenkiste et al., 2006).

Although the self-impact mindset did not differentiate from the social-impact mindset in terms of study engagement, they do when we compare them to the high-impact mindset. The high-impact mindset students reported to have more engagement toward their studies than the self-impact mindset students. At the same time, results also showed that the social-impact mindset and high-impact mindset have similar levels of purpose and study engagement. Both the high-impact mindset and social-impact mindset endorse high self-transcendent and high self-oriented, interest-driven motives for studying. The contrast between these motivational mindsets is based on their (high vs. low) extrinsic reasons for going to university (Hudig et al., 2020). Thus, in addition to strong intrinsic reasons, the extrinsic motives seem to add little value to feelings of purpose and engagement. Yet in the case of self-impact mindset students, strong extrinsic motives with average co-occurring intrinsic, self-oriented motives seem to drive their study engagement. These findings indicate how the interaction between motivational dimensions can bring about a more nuanced perspective in student differences. Future research could clarify whether these levels of purpose and study engagement develop differently during the first academic year and, more importantly, the extent to which these motivational mindsets perform differently and experience different levels of wellbeing over even longer periods of time.

### Purpose in Relation to Study Engagement and the MMM

The results demonstrated that students who reported a stronger sense of purpose also reported more study engagement. This finding supports our suggestion that students become more engaged when they have a sense of purpose in life. Students will invest more time and energy into learning activities if they find their schoolwork meaningful and when they understand the value and relevance of their studies. Future research could further test the directional hypothesis that purpose leads to study engagement. Previous work has indicated that purpose bolsters engagement in high-school settings (Burrow et al., 2018; Hill et al., 2018). This is to the best of our knowledge the first study that explicitly observed the positive association between sense of purpose and study engagement in a university context. We did not identify significant differences in the purpose-engagement relationship across the four mindsets. The relation is thus independent of students’ mindset and the consistency across the motivational mindsets contributes to the validity of the MMM and the MCT.

### Gender and Ethnicity in the MMM

First, it is noteworthy that we identified a pattern in the distribution of gender subgroups across the four mindsets. In our sample, female students were more inclined to have a high-impact mindset or social-impact mindset, while male students were overrepresented in the self-impact mindset and low-impact mindset. As we did not statically test these differences, future research could explore this pattern more in depth.

Further exploration into gender and ethnic differences revealed more interesting patterns. In the overall sample, female students reported both a stronger sense of purpose and more study engagement than male students. This result aligns with earlier findings on gender differences in purpose (Martinez and Dukes, 1997) and in study engagement (Upadyaya and Salmela-Aro, 2015). Probing more deeply into the four mindsets, this gender difference in sense of purpose was, however, only replicated in the self-impact mindset. Likewise, the gender difference in study engagement was only replicated in the high-impact mindset. With respect to ethnicity, a significant effect was identified in study engagement but not in purpose. Minority students reported more study engagement at the beginning of their studies than majority students. This finding among first-year business university students provides further insight into ethnic differences in engagement. Other research, for instance, did not find differences in academic engagement between minority and majority students (Richardson, 2011). What is more remarkable though, is that again only in one of the motivational mindsets, namely, the self-impact mindset, was this ethnic difference in study engagement replicated.

In sum, the exploration into gender and ethnicity provided meaningful patterns in student differences to the extent that they validate the operationalization of the MMM. The differences between subgroups that were identified in the overall sample existed specifically in one type of mindset. These findings suggest that students’ motivational mindset is more important than gender and ethnicity for explaining student differences. The findings indicate that it only matters to which gender or ethnic subgroup you belong if you have a specific mindset. Future research should further explore this and test whether the motivational mindsets and the corresponding levels of purpose and study engagement are better predictors of study success than gender or ethnicity.

### Theoretical and Practical Implications

This study contributes to the literature in several ways. First, by providing novel insight into differences in academic engagement specifically at the beginning of the university program. Although we avoid making causal statements, both students’ motivational mindset and their sense of purpose are associated with the engagement with which students start their studies. Future research could consider and test the role of these resources as potential antecedents of study engagement.

The present study also contributes to the literature by extending the discussion on gender and ethnic differences. While these are exploratory findings, we believe they could be fruitful to explore more deeply in order to better understand gender and ethnic differences in study success. Moreover, the indication that mindset matters more to explain student differences than gender or ethnicity is an important finding because it could potentially enable educators to better design and tailor interventions. After all, educators cannot transform gender or ethnic characteristics; students’ frame of mind, however, is supposedly malleable.

Above all, the preliminary expectations we had regarding the differences in purpose and study engagement across the motivational mindsets were well reflected in the findings of this study. We provided evidence for the predictive validity of the MMM and the classification tool and gained a fuller understanding of each motivational mindset. Students with a high-impact mindset and social-impact mindset showed the most optimal patterns of purpose and study engagement. Both these motivational mindsets are characterized by having a solid combination of self-transcendent and self-oriented, intrinsic motives. Teaching students particularly about the self-transcendent dimension, and guiding them to adopt such study motives, can be highly beneficial to foster their wellbeing (Yeager et al., 2014; Dahl et al., 2020). The self-impact mindset had a weaker sense of purpose than the high-impact and social-impact mindset, while these students reported equal feelings of engagement toward their studies as the social-impact mindset students. The low-impact mindset students seem most prone to experience negative spirals stemming from low engagement at the start of the program (Masten et al., 2005). Future studies should investigate whether these students are more likely to drop out from their studies. Moreover, it requires exploration whether interventions could be targeted specifically to these students to prevent such negative spirals (Upadyaya and Salmela-Aro, 2015). The motivational mindset with which one starts studying could be pivotal for success in the study program. Hence, the MMM and the MCT might help practitioners to recognize early which students have a low-impact mindset and are potentially in the danger zone. These students can then be helped much more rapidly in the process of their studies and instead of drifting aimlessly, they can be supported to flourish and fulfill their potential.

### Strengths, Limitations, and Future Research

The current study is not without limitations. First, quite a number of participants (i.e., 104 students) had to be removed from the sample due to data regulation policies and we cannot test how this has impacted the results. Second, we used a full cohort of business university students. Due to the homogeneous character of the sample, future studies should replicate the findings of this study in other and more diverse samples. Importantly, given the cross-sectional nature of our study, we cannot make any causal claims. Future studies should assess the purpose-engagement relationship longitudinally, test the differences between mindsets over time, and investigate the trajectories of change or stability within students’ motivational mindset. Future research could also collect data through teachers or parents in addition to self-reports to measure students’ motives, sense of purpose, and study engagement. The predictive validity of the MMM for study success has yet to be demonstrated. Future research on the motivational mindsets should therefore include objective performance measures. In the current paper, study engagement was adopted as a single dimension. Future studies could therefore investigate the potential three-dimensional structure of this construct and perform additional analyses. Finally, the sample sizes of the ethnic subgroups within mindsets were quite small. The effect sizes that we found might have been more convincing if we had larger groups of ethnic minority students.

Despite the limitations, we wish to highlight the strengths of the present study. Building on recent person-centered research (Hudig et al., 2020), this study conducted rigorous procedures to validate the novel MMM and the newly developed classification instrument. Findings meaningfully expand the characteristics of the four motivational mindset profiles by including sense of purpose and study engagement. Both sense of purpose and study engagement are developmental assets for students and core elements of student wellbeing. The model and the instrument have been developed from the view that a unidimensional approach to student motivation is insufficient. A multidimensional approach is necessary to understand reality more fully and the findings of this paper enrich that perspective. Future research surely has to further investigate the practical significance of the differences between motivational mindsets, for instance in collaboration with teachers, study advisors, and policy makers. This paper studied a large, representative sample of the student population despite the group of students who refused to process their data. Also, this research was conducted at the very beginning of the study program. Gaining insight into the psychology of students at such a crucial point in their lives may be particularly helpful to prevent negative spirals and foster their flourishing.

## Conclusion

This study produced evidence for the validity of the MMM and the associated MCT. Three exploratory validation procedures were conducted which enhanced the theoretical underpinnings and practical usability of this novel multidimensional perspective of students in higher education. Importantly, our paper showed that the motivational mindsets differ meaningfully in sense of purpose and study engagement, which are two central dimensions of student wellbeing and predictors of study success. This paper is therefore valuable for researchers and practitioners as it provides new insight into differences in student wellbeing specifically when students enter the university program. Students with a high-impact mindset and social-impact mindset showed the most optimal patterns in these student qualities, while the low-impact mindset students revealed the least optimal pattern in sense of purpose and study engagement. By means of the MCT, low-impact mindset students can be recognized as early as possible in order to support their wellbeing and study career. The data presented here suggest that educational practitioners can promote a shift in thinking toward self-transcendent motives to cultivate students’ wellbeing and potentially improve their study success.

## Data Availability Statement

The raw data supporting the conclusions of this article will be made available by the authors, without undue reservation to any qualified researcher.

## Ethics Statement

The studies involving human participants were reviewed and approved by Internal Review Board of the Erasmus Research Institute of Management, Erasmus University Rotterdam. The patients/participants provided their written informed consent to participate in this study.

## Author Contributions

JH concepted and designed the study, performed the statistical analysis, and has written the draft of the manuscript. AS assisted in performing the statistical analysis. AS, MS, and GS contributed to the conception and design of the study. All authors contributed to revise, read and approve the submitted version of the manuscript.

## Conflict of Interest

The authors declare that the research was conducted in the absence of any commercial or financial relationships that could be construed as a potential conflict of interest.

## Publisher’s Note

All claims expressed in this article are solely those of the authors and do not necessarily represent those of their affiliated organizations, or those of the publisher, the editors and the reviewers. Any product that may be evaluated in this article, or claim that may be made by its manufacturer, is not guaranteed or endorsed by the publisher.

## Footnotes

^1^We distinguish between ethnic majority (i.e., Dutch) and ethnic minority students. Western minority students were excluded from comparing analyses. Following the definition of Alders (2001, p. 2) who stated that “ethnic minorities” are individuals with a first- and second-generation foreign (non-Dutch) background. “First generation consists of persons who are born abroad and have at least one parent who is also born abroad. The second generation consists of persons who are born in Netherlands and have at least one parent who belongs to the first generation.”

^2^Western minority students (i.e., 47 participants or 7.1%) were excluded from the analysis of RQ3b because we focused specifically on comparing the non-western ethnic minority students and Dutch majority students.
